# Oral Baclofen Toxicity in a Pediatric Patient Managed Without Invasive Intervention: A Case Report and Literature Review

**DOI:** 10.7759/cureus.101812

**Published:** 2026-01-18

**Authors:** Bandar Alqahtani, Muhammad N Nizamuddin, Syed Rayees

**Affiliations:** 1 Department of Emergency Medicine, Armed Forces Hospital Southern Region, Khamis Mushait, SAU; 2 Department of Pediatrics, Armed Forces Hospital Southern Region, Khamis Mushait, SAU

**Keywords:** baclofen toxicity, brain death mimicking, muscle spasticity, pediatric case, toxicology and poisoning

## Abstract

Baclofen is utilized for the management of spasticity associated with neurological disorders such as cerebral palsy, multiple sclerosis, and spinal cord injuries. It acts as an agonist for the gamma-aminobutyric acid (GABA)-B receptor. Although baclofen proves to be effective, it presents a significant risk of toxicity and withdrawal symptoms, particularly among pediatric patients, where pharmacokinetics and clinical presentations may diverge from those observed in adults. Furthermore, baclofen is generally not detected in standard toxicology screening, underscoring the need for heightened clinical awareness. We report a case involving a five-year-old boy who inadvertently received a toxic dose of baclofen. He was admitted to the Pediatric Intensive Care Unit via the pediatric emergency room, exhibiting signs and symptoms of convulsions, drowsiness, and bradycardia. The convulsions were managed with intravenous diazepam, and the child made a full recovery within 24 hours with appropriate supportive care. This case report emphasizes that prompt intervention in the case of baclofen overdose prevented the need for invasive procedures such as mechanical ventilation and hemodialysis. Effective supportive therapy yields favorable outcomes, given that there has been no hypoxic or ischemic injury before receiving medical assistance. This literature review focuses on oral baclofen toxicity, without delving deeply into the topics of intrathecal baclofen toxicity and withdrawal. This case contributes to the expanding literature on pediatric baclofen toxicity and highlights the potential for favorable outcomes with prompt intervention.

## Introduction

Baclofen is a skeletal muscle relaxant and gamma-aminobutyric acid (GABA)-B receptor agonist used to alleviate spasticity in conditions like multiple sclerosis, spinal cord injuries, and cerebral palsy, the latter of which is one of the most common childhood-onset neurological disorders. It is also used off-label for alcohol withdrawal, gastroesophageal reflux disease, muscle spasm, and chronic hiccups [1,2]. Despite widespread use, evidence for baclofen's clinical efficacy remains inconsistent, and its use carries the risk of severe toxicity and withdrawal syndromes, necessitating high clinical vigilance. Clinically, baclofen reduces muscle stiffness, spasticity, and pain, thereby improving movement, promoting rehabilitation, and enhancing overall quality of life [2-4]. However, misuse and overdose, particularly among adolescents, can lead to symptoms even mimicking brain death, underscoring the importance of early recognition and appropriate management [5-7]. Another diagnostic challenge lies in the fact that standard drug screening panels do not detect baclofen. This limitation necessitates a high index of suspicion based on clinical presentation and history. Laboratory confirmation often requires specific assays that may not be readily available in all healthcare settings, especially in resource-limited regions [8,9]. Similarly, at our hospital, baclofen drug levels were not available to confirm the diagnosis; however, a clear history of baclofen overdose allowed us to establish the diagnosis confidently. Management of baclofen toxicity remains supportive, with airway and hemodynamic stabilization as cornerstones of care, and mechanical ventilation and hemodialysis added when needed [10-12].

## Case presentation

We present a case involving a five-year-old boy with diplegic cerebral palsy who was brought to the Emergency Department three hours after ingesting a toxic dose of baclofen at his residence. At home, he was drowsy and unresponsive after the ingestion, subsequently developing one episode of generalized tonic-clonic convulsion. Upon his arrival at the emergency department, he was found to be in a postictal state with a Glasgow Coma Scale score of 9/15 (E2 V3 M4). The patient was afebrile, drooling with frothy sputum, and had constricted pupils. Chest, cardiac, and abdominal examinations were unremarkable. There were bilateral lower limb casts. He had bradycardia with a heart rate of 50-60 bpm, fluctuating blood pressure (systolic 102-119 mmHg, diastolic 47-75 mmHg), a respiratory rate of 25 breaths per minute with shallow, spontaneous respirations, and oxygen saturation of 100% on face-mask oxygen at 5 L/min. Blood glucose was 6.4 mmol/L.

The child had undergone bilateral lower limb soft tissue contracture release surgery for a short Achilles tendon the same morning and was discharged on oral baclofen syrup (10 mg/mL), prescribed at a dose of 2.5 mg (0.25 mL) every 12 hours. However, he accidentally received 15 mL (150 mg) instead of 0.25 mL at home a few hours after discharge, due to misinterpretation of the dose written on the medicine cover. This was the first time he was prescribed baclofen. Routine toxicology screening was negative. Serum baclofen levels were unavailable at our hospital.

The child was delivered through an emergency cesarean section at 38 weeks due to the presence of placenta previa. There was no documentation of NICU admission, and he was discharged after two days of monitoring. By the time he turned one, he had successfully reached his developmental milestones. However, there was a delay in his walking, which started at 18 months, with a tip-toe style. The development of an atypical gait followed this. At the age of two, he underwent an MRI brain scan, which revealed bilateral, nearly symmetrical abnormal white matter signal changes, suggestive of periventricular leukomalacia because of hypoxic-ischemic brain injury, eventually leading to a diagnosis of cerebral palsy. During this time, he was also receiving consistent physiotherapy sessions focused on muscle relaxation and mobility training.

In the emergency department, his airway patency was assessed and maintained, high-flow oxygen therapy was continued with pulse oximetry monitoring, and intravenous access was secured. IV fluids were started, keeping the patient nil per os (NPO). Shortly after arrival, the child developed a second episode of generalized tonic-clonic convulsion, which was promptly aborted with intravenous diazepam (single dose). An endotracheal tube was kept ready throughout supportive management in the ER for potential mechanical ventilation if respiratory compromise occurred. Postictally, he remained drowsy but maintained adequate spontaneous respiration.

Given the history and clinical picture of baclofen toxicity with altered mental status and seizures, the pediatric intensive care unit team and toxicology team were alerted. The toxicology team advised keeping the patient in the PICU for close observation, as baclofen toxicity does not have any antidote. Basic laboratory investigations, including renal and liver function tests, electrolytes, blood gas analysis, and complete blood count, were ordered. An ECG and chest X-ray were also performed to rule out cardiopulmonary complications. ECG showed sinus bradycardia. The patient was subsequently transferred to the PICU for supportive management and close monitoring, with mechanical ventilation kept on standby in anticipation of possible respiratory depression. Basic laboratory test results were within normal limits, as shown in Table 1. Venous blood gas analysis was unremarkable (pH=7.35, pCO_2_=4.8 kPa, pO_2_=10.3 kPa, HCO_3_=20.9 mmol/L).

**Table 1:** Laboratory parameters

Lab Parameter	Measured Value	Reference Range
Hemoglobin	11.53 g/dL	10.9–15 g/dL
Total leukocyte count	8.07 × 10⁹/L	6–14 x10^9^/L
Platelet	386 × 10⁹/L	140–440 x10^9^/L
C-reactive protein	2.0 mg/L	<6 mg/L
Serum creatinine	38.8 µmol/L	27–53 µmol/L
Sodium	139 mmol/L	131–145 mmol/L
Potassium	4.2 mmol/L	3.6–6 mmol/L
Direct bilirubin	5.0 µmol/L	1.7–8.6 µmol/L
Alanine aminotransferase (ALT)	14.0 U/L	8–36 U/L
Aspartate aminotransferase (AST)	38 U/L	13–38 U/L

His health steadily improved, with stable vital signs, and he regained full consciousness within 24 hours with supportive care only. He was discharged on the fifth day and scheduled for follow-up appointments in the orthopedics and pediatric neurology clinics. The parents and caregivers were provided with education and training on the importance of precise dosing, proper storage, and appropriate timing for medication administration.

## Discussion

Baclofen acts as a GABA-B receptor agonist, distinguishing it from other CNS depressants such as benzodiazepines and barbiturates, which target GABA-A receptors. It exerts both presynaptic and postsynaptic inhibitory effects. The presynaptic inhibitory effect of baclofen prevents calcium influx, thereby reducing GABA release. In overdose, this can paradoxically increase seizure risk [1,2]. Postsynaptic inhibition of baclofen increases potassium efflux, thereby enhancing neuronal inhibition. Sudden withdrawal can also lower the seizure threshold. These mechanisms explain the paradoxical presentation of seizures in both overdose and withdrawal scenarios. Other neurons in the body, such as those in the central and sympathetic nervous systems, have GABA-B receptors, which may explain the adverse effects of sleepiness and lightheadedness [2,3].

Initially developed in the 1960s as an antiepileptic, baclofen was reintroduced in 1971 for the treatment of spasticity [2,4]. It acts by mimicking GABA and binding to GABA-B receptors in the brain, spinal cord, and peripheral tissues, thereby inhibiting nerve transmission and reducing muscle spasm [5-7]. Baclofen is administered either orally or intrathecally; in adults, oral doses typically start at 5 mg three times daily and are titrated to a maximum of 80 mg/day. Oral doses, in children aged two to seven years, typically start at 10-15 mg/day divided every eight hours and titrated every three days by 5-15 mg/day to a maximum of 40 mg/day, and in children aged eight years and above, titrated similarly to a maximum of 60 mg/day. Intrathecal doses range from 50 to 1000 mcg/day [8-10]. Oral baclofen has 70-85% bioavailability but limited CNS penetration (possible at high doses) due to its moderate lipophilicity; the kidneys primarily excrete it with 60-80% of the drug in its unchanged form. Around 15% undergoes hepatic metabolism. It has a half-life of approximately two to six hours, with a rapid onset of action. Peak serum levels are reached between 45 minutes and two hours. The half-life after intrathecal administration is less than five hours, with onset of action from 30 minutes to one hour, and peak serum levels are reached at around four hours [9,11].

Managing reversible spasticity brought on by multiple sclerosis and spinal cord injuries is one of the oral indications of baclofen, while treatment of severe spasticity brought on by multiple sclerosis and spinal cord damage is one of the intrathecal indications. Baclofen can be used for cerebral causes of spasticity, such as cerebral palsy and traumatic brain damage, in patients older than four [2,12-14]. With the expansion of baclofen's use beyond its traditional purpose of managing spasticity, there has been a corresponding increase in cases of overdose and toxicity, highlighting the critical need for vigilance in its monitoring. Some of the off-label uses for baclofen include musculoskeletal pain/spasm, alcohol use disorder, gastroesophageal reflux disease, chronic hiccups, chronic post-traumatic stress disorder, narcolepsy, trigeminal neuralgia, and chronic low back pain, among others [13,14].

Baclofen overdose presents a broad spectrum of clinical manifestations ranging from mild lethargy to deep coma and respiratory failure. It is frequently associated with polypharmacy or recreational misuse, particularly among adolescents and young adults [1,2,12], and remains a potentially dangerous drug when taken inappropriately or at excessive doses. Adults often experience severe toxicity above 200 mg/mL, with therapeutic serum values ranging from 80 to 400 mg/mL. Nevertheless, there is no discernible relationship between dosage and symptom intensity, or between dosage and the need for ventilation, in children [15,16].

Symptoms of toxicity usually occur within 30 to 120 minutes post-ingestion but may be delayed. Symptoms vary based on dose, patient age, renal function, and concurrent medications [12,13]. In various situations, especially among pediatric patients or in contexts where a reliable medication history is not available, baclofen toxicity can often be confused with other neurological issues. It is imperative to note that the severe CNS depression and muscle flaccidity resulting from an overdose can imitate brain death, which makes it vital for clinicians to recognize the toxidrome to avoid premature or incorrect assessments. Still, full recovery is possible with adequate support [1-3,5,13-17]. Seizures are common because baclofen is a proconvulsant, but they are usually short-lived and treatable. While spasticity and hyperreflexia are more frequently experienced with baclofen withdrawal, hypotonia and flaccid paralysis should be taken into consideration when considering baclofen poisoning. The complications of baclofen toxicity are significant and can lead to fatal outcomes; thus, early detection and a strong level of suspicion are imperative. Clinical signs and symptoms of baclofen poisoning include lethargy, coma, agitation, ataxia, hyporeflexia, hypotonia, seizures, nonconvulsive status epilepticus, and respiratory depression and failure, often requiring mechanical ventilation. Hemodynamically, it can cause bradycardia, hypotension, tachycardia, and hypertension. A few autonomic signs include hypothermia, mydriasis or miosis, urinary retention, nausea, and vomiting [14,15].

The diagnosis of baclofen toxicity primarily relies on clinical observations, supported by the patient's history and symptoms. Healthcare providers need to be especially alert, as symptoms can vary widely in presentation and severity. Clinical suspicion plays a crucial role in toddlers or individuals exhibiting sudden, unexplained central nervous system depression [1,2,4,6]. The toxicology screen has limitations for baclofen, as its levels are not typically available. It is vital to check electrolytes and glucose to spot any metabolic issues or hypoglycemia that could be alternative or contributing factors. Assessing renal function is necessary to determine how well the drug is cleared, as the kidneys primarily excrete baclofen [6,7]. A blood gas test is needed to assess respiratory status and detect respiratory acidosis. Neuroimaging or a lumbar puncture should be done to rule out other causes, such as infection or intracranial pathology [6,8]. If there is a prolonged altered mental status, an electroencephalogram is needed to check for non-convulsive seizures. In children, especially those with cerebral palsy, baclofen can be absorbed differently in the gastrointestinal tract and may have a short half-life of about 4.5 hours [18].

The management of baclofen toxicity is mainly supportive: ensuring airway protection and providing mechanical ventilation in cases of respiratory depression. Benzodiazepines should be administered for agitation or seizures. Additionally, fluids and vasopressors are necessary for addressing hypotension [1,2,12,13]. A dose exceeding 5 mg/kg warrants gastric decontamination; activated charcoal may be utilized if the patient arrives within one hour of ingestion and the airway is secured [13]. There is no specific antidote; however, physostigmine may aid with central side effects such as somnolence and respiratory depression, although its efficacy varies. Bradycardia, breathing, temperature regulation, and cardiac output may all be improved by intravenous atropine. Hemodialysis is effective in lowering baclofen levels in patients with renal impairment and has also demonstrated benefits in certain patients with normal renal function, particularly those with prolonged, severe symptoms [17-19].

The existing literature concerning pediatric cases of oral baclofen toxicity is limited, whereas a greater number of toxicity cases have been documented with the intrathecal administration. Table 2 below summarizes the published research on pediatric oral baclofen toxicity to date. It suggests that, in pediatric cases, the toxic dose does not correlate with the need for mechanical intubation or dialysis. Instead, it varies on a case-by-case basis. However, it simultaneously emphasizes the importance of promptly assessing potential toxicity to manage conservatively, thereby ensuring a favorable prognosis without the need for further interventions that arise from diagnostic delays. This also underscores the importance of dose modifications, or total avoidance, in patients with renal impairment, irrespective of their age, to avert unforeseen accumulation and neurotoxic effects. Furthermore, there is a necessity for educating caregivers about medication safety and dosing and appropriate storage practices to mitigate the risk of accidental poisoning.

**Table 2:** A brief summary of case reports published about oral baclofen toxicity and their outcomes

Case Reports	Age	Symptoms on Presentation	Kidney Function	Mechanical Ventilation Required	Hemodialysis Required	Baclofen Dose	Baclofen Level in Plasma
Shah SA et al., 2020 [1]	6-year-old with cerebral palsy, neuromyelitis optica	Deeply comatose	Normal	Yes	Yes, two sessions	1300 mg	4.00 mcg/mL (normal reference range 0.08–0.4 mcg/mL)
Syafruddin et al., 2025 [8]	2-year-old	GCS 6/15 Drowsy, bradypneic, bradycardic, hypotensive, hypothermic with constricted unreactive pupils, generalized hypotonia, and areflexia	Normal	Yes, 19 hours	No	An estimated 300 mg	Not measured
Marcellus C et al., 2019 [9]	4-year-old healthy	Deeply comatose, miotic pupils	Normal	Yes, 36 hours	No	Unknown	2009 ng/mL (normal therapeutic range for neurological disorders is 100–400 ng/mL)
Mishaal et al., 2020 [10]	2½-year-old	Decreased consciousness, bradypnea, bradycardia	Acute kidney injury on peritoneal dialysis	No	No	15 mg total	Not measured
	9-year-old healthy	Decreased consciousness, generalized decreased tone with upper airway obstruction, increased work of breathing,	Toxic shock syndrome leading to severe AKI, requiring CRRT, then PD	Nasopharyngeal airway	No	20 mg total	Not measured
Dasarwar et al., 2009 [13]	3-year-old healthy	GCS 3/15 Deeply comatose, bradycardia, hypotension, hypotonia	Normal	Not mentioned	Yes	Six 10 mg tablets	Not measured
Chapple et al., 2001 [14]	1-year-old	Hypotonia, hyporeflexia, ataxia, severe respiratory depression	Normal	Yes, 10 hours	No	Unknown	1502 ng/mL
	4-year-old			Yes, 13 hours		150 mg	708 ng/mL
Dasgupta K et al., 2009 [16]	2-year-old-girl healthy	Deeply comatose, hypotonia	Normal	Yes	No	Unknown	Not measured
Zaki SA et al., 2014 [17]	10-month-old	GCS 3/15 Deeply comatose	Normal	Not mentioned	No	Six 5 mg tablets	Not measured
Malak M et al., 2015 [18]	6-year-old boy	GCS 10/15 Decreased consciousness, hypotonia, areflexia, mydriasis	Advanced renal failure	No	No	20 mg	Not measured
Skiles CMF et al., 2021 [19]	2-year-old	Drowsiness, lethargy, low muscle tone	End-stage kidney disease	No	Peritoneal dialysis	0.25 mg/kg/day, divided three times daily for 1 day	0.55 mcg/mL

## Conclusions

Baclofen toxicity represents a critical clinical issue that can lead to complete recovery when managed correctly. In the current case, the patient achieved full recovery through intensive supportive care, without the need for mechanical ventilation or hemodialysis. It is crucial to identify its unique clinical presentation, particularly in pediatric patients, to ensure prompt diagnosis and treatment. Future research should prioritize large-scale, multicenter studies to investigate the incidence, clinical spectrum, and outcomes of baclofen toxicity, particularly in pediatric populations. Creating standardized treatment protocols and raising awareness among healthcare providers are vital actions to enhance patient outcomes. Developing a comprehensive program to educate caregivers on medication safety, drawing attention to the risks of accidental intake, possible side effects, and the importance of secure storage, is paramount.
